# Medicine as a Profession

**Published:** 1896-09-05

**Authors:** 


### MEDICINE AS A PROFESSION.

The choice of a profession is a matter which every year becomes more serious and in a sense more difficult, for not only has competition grown to such proportions that the prospect of earning even a living has become less of a certainty, but the time, the labour and the money which must be spent before any return can be expected, have of late years so increased that the loss in case of failure becomes a very serious matter. And in medicine failure, complete and absolute, is far more common than many people imagine. Anyone who will take the trouble to ascertain the number of candidates who fail at every examination, and will bear in mind with what slender resources many men enter upon the study of medicine, will quickly see what indeed is a well recognised fact, that a not inconsiderable proportion of those who register as medical students fail to register as qualified medical practitioners. Even, however, when they have succeeded in passing all examinations and obtaining their diplomas, each year's batch of newly-made practitioners find themselves cast out upon a world which is less and less anxious to receive them. Competition is by no means limited to the years of study. From the moment when the young man is qualified and, with the necessity of earning his living before him, finds that the question he has to face is not how to cure patients but how to get them, to the time when, as an old man at fifty, he finds himself pushed aside by younger men, with nimbler limbs and wits more primed with novelties, competition is everywhere present throughout the doctor's life; an incentive to hard work in days when health is good; a spur to ambition; often a severe temptation to do what is wrong—or, at least, what is ethically incorrect—for the sake of filthy lucre; and then, as strength begins to fail and responsibilities increase, this ever-present competition becomes a perpetual nightmare, and when the doctor finds time to look into the future he finds it painted in blank hopelessness.

Connected with the medical profession are certain charities which give some little help to those of its members who have suffered the buffettings of fortune. These societies work secretly, and tell no man what they do. Outsiders know nothing of the straits to which good men, and men of good professional position, are at times reduced; but the officials of these societies are well aware how hard is the doctor's life, how difficult it is to make ends meet, and how often it happens that slight illnesses, the smallest interruptions to the daily round, bring apparently prosperous medical men to the verge of want and penury. Perhaps if these charities worked more publicly, and if people understood how much more frequently it is the doctor's lot to eat the bread of carefulness and die unknown than it is to meet with wealth and die in honour, the number of those who crowd into the profession year by year would show some sign of diminution. As it is, competition, cruel and unrestrained competition, each man playing for his own bat, with but little thought for others so that he may but win fame, or even notoriety, seems the order of the day; and so it happens that unless a man have money, by dint of which he can afford to wait until practice comes, or unless he have such large supply of wit as to be able to force the hand of fortune, his chances of real success in medicine are but small. Better keep a shop at once than lead the lives that some men do.

